# An Inexpensive Co-Intercalated Layered Double Hydroxide Composite with Electron Donor-Acceptor Character for Photoelectrochemical Water Splitting

**DOI:** 10.1038/srep12170

**Published:** 2015-07-15

**Authors:** Shufang Zheng, Jun Lu, Dongpeng Yan, Yumei Qin, Hailong Li, David G. Evans, Xue Duan

**Affiliations:** 1 State Key Laboratory of Chemical Resource Engineering, Beijing University of Chemical Technology, 15 Beisanhuan East Road, P. Box 98, 100029, Beijing (P. R. China); 2 College of Chemistry, Beijing Normal University, 19 Xinjiekou Outside Street, 100875, Beijing (P. R. China); 3 Beijing Engineering Center for Hierarchical Catalysts, Beijing University of Chemical Technology,15 Beisanhuan East Road, P. Box 98, 100029, Beijing (P. R. China)

## Abstract

In this paper, the inexpensive 4,4-diaminostilbene-2,2-disulfonate (DAS) and 4,4-dinitro-stilbene-2,2- disulfonate (DNS) anions with arbitrary molar ratios were successfully co-intercalated into Zn_2_Al-layered double hydroxides (LDHs). The DAS(50%)-DNS/LDHs composite exhibited the broad UV-visible light absorption and fluorescence quenching, which was a direct indication of photo-induced electron transfer (PET) process between the intercalated DAS (donor) and DNS (acceptor) anions. This was confirmed by the matched HOMO/LUMO energy levels alignment of the intercalated DAS and DNS anions, which was also compatible for water splitting. The DAS(50%)-DNS/LDHs composite was fabricated as the photoanode and Pt as the cathode. Under the UV-visible light illumination, the enhanced photo-generated current (4.67 mA/cm^2^ at 0.8 V vs. SCE) was generated in the external circuit, and the photoelectrochemical water split was realized. Furthermore, this photoelectrochemical water splitting performance had excellent crystalline, electrochemical and optical stability. Therefore, this novel inorganic/organic hybrid photoanode exhibited potential application prospect in photoelectrochemical water splitting.

Solar energy has been regarded as the most ideal alternative for fossil energy resource due to its combination of powerful, renewable, affordable, and sustainable properties^1^. Furthermore, efficiently water splitting into applicable hydrogen by solar energy was considered as the ultimate solution for the energy and environment crisis^2^. Consequently, tremendous attention has been focused on water splitting for hydrogen generation by photocatalysis or photoelectrochemical (PEC) process^3^. Since the pioneer work of Honda *et al.* on a TiO_2_ photoanode^4^, the principle of PEC hydrogen production was successfully extended into systems of inorganic semiconductor particulates. However, several key factors, such as low light absorption at visible-light, rapid charge recombination, and instability of semiconductor materials during chemical process, strikingly restricted the realization of large-scale industrial application for this “dreaming technology”^5^. In general, the activity of water photolysis was highly dependent on co-catalyst, and most reaction systems needed the incorporation of other oxidizing or reducing species as sacrificial agents to participate in the reaction. All these work was focused on inorganic semiconductors based on the water reduction/oxidation by photo-excited electron/hole on the band edge.

On the contrary, there existed numerous organic molecules with various HOMO/LUMO electron levels, which could form electron donor-acceptor (D-A) system when in contact^6^. Charge-transfer complex salt with organic electron donor, such as BEDT-TTF^7^ and κ-(BETS)_2_FeBr_4_^8^, showed excellent conductivity and magnetic properties. The D-A pairs based on organic polymers and small molecules semiconductors had been widely used in organic field-effect transistors (OFETs)^9^, organic photoconductor^10^, and D-A type heterojunctions^11^ as organic solar cells (like P3HT/PCBM blends^12^, C_70_/zinc phthalocyanine^13^). In these D-A systems, the photo-induced electron transfer (PET) process across a D-A interface was the key process, which dominated the efficiency of photoelectric conversion^14^. However, the D-A organic systems were rarely applied into the PEC water splitting, maybe due to the expensive price of D-A molecules and the photo-instability of most organic molecules. Toshiyuki *et al.*^15^ recently reported that the organic PTCBI/CoPc bilayer could act as a stable photoanode that was capable of evolving O_2_ at the CoPc/water interface. Therefore, it was necessary to study the novel inexpensive and photo-stable D-A system to effectively realize the PEC water splitting.

4,4-diaminostilbene-2,2-disulfonic acid (DAS) and 4,4-dinitro-stilbene-2,2-disulfonic acid (DNS) are cheap industry intermediates, and have been widely used in the production of optical brighteners and synthetic dyes^16^. The nitro/amino group substitution lowered/raised the electron energy levels of DNS/DAS, respectively, compared to the unsubstituted parent^17^. Therefore, it could be expected that DAS and DNS molecules could be designed as electron D-A system upon mixing. In order to immobilize this D-A molecular system in solid state to facilitate their PET effect, Zn_2_Al layered double hydroxides (LDHs), [Zn_2_Al(OH)_2_](NO_3_)·*m*H_2_O)^18^, with versatility in terms of their chemical composition, layer charge, and capability of ion exchange was selected as the host layers. DAS and DNS anions could be co-intercalated into the 2D interlayers of the LDHs layers to create their close contact, which was the primary condition for the PET process. Furthermore, the co-intercalated system was fabricated into the thin film which realized the broad and strong optical absorption and fluorescence quenching, implying the obvious PET process between the intercalated DNS and DAS anions. The DAS and DNS co-intercalated Zn_2_Al-LDHs thin film photoanode exhibited excellent abilities of PEC water splitting. This electron D-A type 2D co-intercalation composite was unique due to the following points, i) the positive-charged host layers will affect the photoexcited electron/hole, which was favorable for the charge separation during the PET process; ii) the interlayer water molecules adsorbed on the LDHs nanosheets would be accessible for the photolysis; iii) the energy levels of photoexcited electron/hole could be apt to modify by various co-intercalation design; iv) in contrast with the band edge absorption of inorganic semiconductor, this 2D composite possessed the broad and strong optical absorption in the UV-visible region, due to the organic π*-π electron transition of guest anion, in favor of the absorption of solar light; v) the intercalation of organic molecules into Zn_2_Al-LDHs interlayers could improve the photo/thermostability of composites due to the UV-shield effect of host layers^19^.

## Results

### Characterizations of DAS(*x*%)-DNS/LDHs

**Figure 1:**
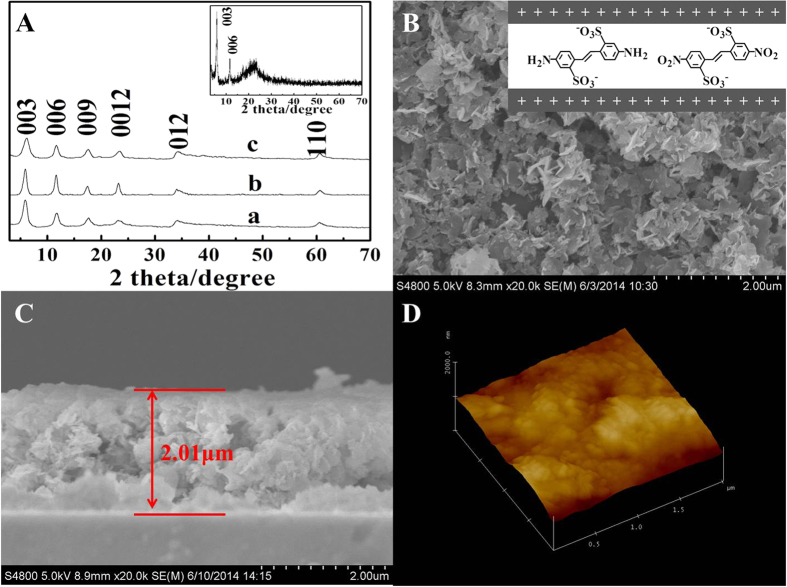
The XRD patterns of the as-prepared samples (**A**) a-DNS/LDHs, b-DAS(50%)-DNS/LDHs, c-DAS/LDHs), Inset: DAS(50%)-DNS/LDHs thin film; (**B**) Top and (**C**) side-view SEM images and (**D**) tapping-mode AFM image of the DAS(50%)-DNS/LDHs thin film.

DAS/DNS co-intercalated Zn_2_Al-LDHs powder was prepared by hydrothermal co-precipitation method^20^, and was nominated as DAS(*x*%)-DNS/LDHs; *x* is the molar percentage of DAS account for all interlayered anions. It was found that the DNS and DAS anions could be co-intercalated into the interlayers of Zn_2_Al-LDHs with arbitrary ratio (*x* = 0 ~ 100). Interestingly, the 003 diffraction peaks of DAS(*x*%)-DNS/LDHs were almost at the same position due to the similar shape of DAS and DNS anions (Fig. 1A, Fig. S1 in SI). The layer spacing was varied from 1.44 nm to 1.50 nm (Fig. S2 in SI), which meant that the co-intercalated DAS/DNS anions were monolayered orderly aligned with the electrostatic interaction and π-π stacking effect (Fig. 1B inset), according to the analysis of interlayer spacing (1.5 nm), one LDH layer (0.48 nm) plus the lateral dimension of DAS(0.90 nm)/DNS(0.93 nm) (Fig. S3). The co-intercalation was also confirmed by their FT-IR spectra (Fig. S4, S5). These co-intercalated powders were further spin-casting^21^ into thin films as a photoanode to investigate its PEC properties, which showed an excellent *c* orientation (Fig. 1A inset). The scanning electron microscope (SEM) images (Fig. 1B) exhibited that the surface of DAS(50%)-DNS/LDHs thin film was smooth and continuous, and the average thickness of the film was *ca*. 2.01 μm (Fig. 1C). The lamellar crystallites were stacked with the *ab* plane parallel to the substrate, which was consistent with the result of XRD (Fig. 1A inset). The average root-mean-square (rms) roughness of the film was 25.046 nm, which was inferred from the atomic force microscope (AFM) topographical image (scan = 2 μm × 2 μm) (Fig. 1D).

### Optical properties of DAS(*x*%)-DNS/LDHs

**Figure 2:**
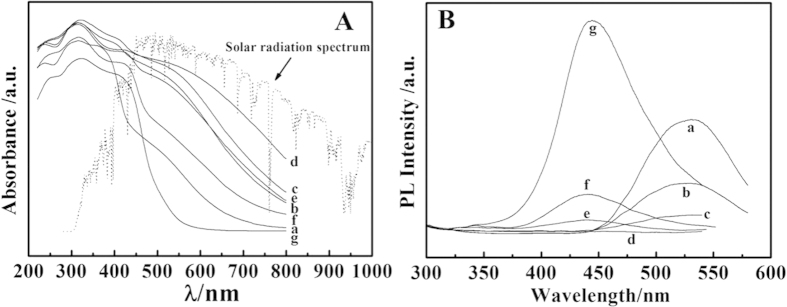
(**A**) The absorption spectra (dot line: the AM1.5 solar spectra) and (**B**) the PL emission spectra of the DAS(*x*%)-DNS/LDHs samples (a) 0%, (b) 10%, (c) 30%, (d) 50%, (e) 70%, (f) 90%, (g) 100% (*λ*_*ex*_ = 280 nm).

It was interesting that the DAS(*x*%)-DNS/LDHs powders showed colors change depending on the DAS content since the Zn_2_Al/LDHs layers were transparent in this spectral region. The color became darker for the ratio approaching to *x* = 50% and was the darkest brown black for *x* = 50% (Fig. S6). Correspondingly, the optical absorption spectra of DAS(*x*%)-DNS/LDHs (Fig. 2A) were dominated by the DAS content, and it showed the broad and strong absorption ranged from 800 nm to 220 nm for *x* = 50%, which matched for the AM1.5 solar radiation spectrum, in contrast with the narrow UV absorption band in the DAS/DNS mixture solution (Fig. S7A), which was attributed to the 2D confined effect of LDHs monolayer, and also in favor of the subsequent PET process between DAS and DNS anions^22^.

This 2D PET process of this co-intercalated composite could also be witnessed by the photoluminescence (PL) spectroscopy. The DAS (10^−3^ M) and DNS (10^−2^ M) aqueous solution were luminous at 426 nm and 530 nm (Fig. S8), respectively, and the PL emission of DAS overlapped with the PL excitation of DNS, which implied that the PET process was possible between DAS and DNS anions. As an energy matched electron D/A pair^17^, when the DAS and DNS anions were mixed together with different ratios, both luminescence were quenched to different extent (Fig. S7B). The least PL emission appeared at the ratio of *x* = 50%, which was a direct indication of PET process between DAS (D) and DNS (A)^23^. Coincidentally, the DAS/LDHs and DNS/LDHs powders were luminous at 445 nm and 530 nm, respectively, being similar with the DAS and DNS solutions. The PL intensity of DAS(*x*%)-DNS/LDHs powders displayed the similar behaviors with DAS/DNS mixed solutions, and both of PL emission were quenched completely when *x* = 50% (Fig. 2B). Therefore, It can be concluded that the similar anionic shape, the special 2D hydrophobic interlayer environment made the co-intercalated DAS and DNS anions close stack, and orderly paralleled align, which was prone to the occurrence of 2D PET process.

### Energy level alignment of DAS/DNS within the LDHs interlayers

The energy level match between electron donor and acceptor was the premise for PET process. The highest occupied molecular orbital (HOMO) and the lowest unoccupied molecular orbital (LUMO) energy levels of the DAS/DNS co-interacted system and pristine DAS/DNS solution were evaluated by the cyclic voltammetry combined with optical absorption/PL spectroscopy (Fig. S9, S10)^24^. The measured energy level values were listed in Table 1. It can be found that the *E*_g_ of intercalated DAS/DNS was narrowed compared to the pristine DAS/DNS anions, and the energy level was decreased on the whole, which may be caused by the 2D confined effect of LDHs. The LUMO/HOMO energy levels for the intercalated DAS and DNS were *ca*.−2.68/−5.50 eV and *ca*.−3.48/−5.82 eV, respectively, both of which indicated that the PET process was energy reasonable for the co-intercalated DAS/DNS anions. Compared with the reversible hydrogen/oxygen electrode potential of H^+^/H_2_ and O_2_/H_2_O, it also revealed that this HOMO/LUMO energy level alignment of DAS(50%)-DNS/LDHs was compatible for PEC water splitting, provided that no remarkable difference of energy levels between the co-intercalated and single-intercalated composite.

### The PEC properties

**Figure 3:**
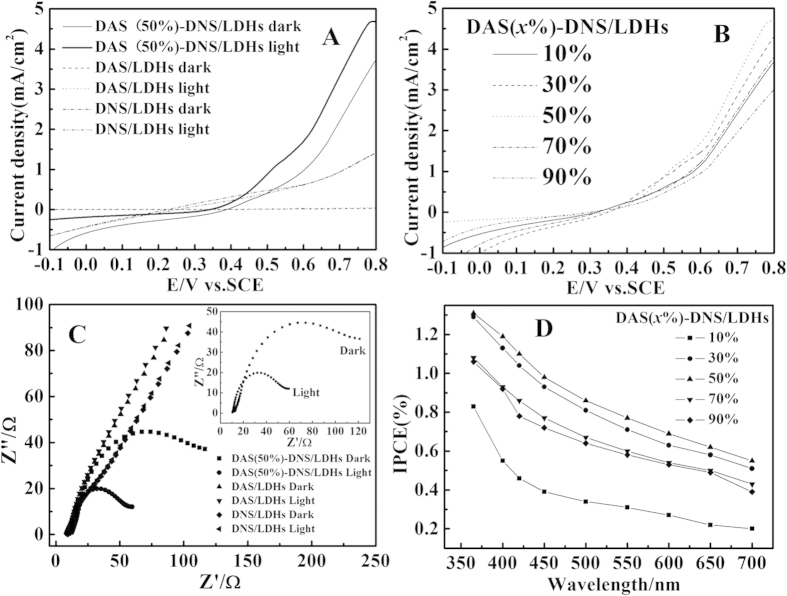
(**A**) Current-voltage curves of DAS/LDHs, DNS/LDHs, and DAS(50%)-DNS/LDHs in dark and in light at a scanning rate of 50 mV·s^−1^; (**B**) Current-voltage curves of DAS(*x*%)-DNS/LDHs in light at a rate of 50 mV·s^−1^; (**C**) The EIS curve measured at 0.8 V vs. SCE of DAS(50%)-DNS/LDHs, DAS/LDHs, DNS/LDHs. Inset: The magnified EIS curve of DAS(50%)-DNS/LDHs; (**D**) The IPCE spectra for DAS(*x*%)-DNS/LDHs at applied voltage of 0.8 V vs. SCE. The light source was 300 W Xe lamp illumination.

The photon-generated current of DAS(50%)-DNS/LDHs photoanode showed the prominent current above the onset voltage (0.4 V vs. SCE) and was remarkably enhanced (4.67 mA/cm^2^ at 0.8 V vs. SCE) under light illumination, compared with its dark current (3.73 mA/cm^2^ at 0.8 V vs. SCE), while the single-intercalated DAS-LDHs or DNS-LDHs photoanode was inert or poor electric conductive in dark or in light (Fig. 3A). This photocurrent reached the maximum for *x* = 50%, compared with other ratios (Fig. 3B). Concomitant with the remarkable photocurrent, hydrogen and oxygen was evolved and bubbled at the Pt cathode and photoanode (Fig. S11), respectively, indicating the PEC water splitting occurring. In the electrochemical impedance spectroscopy (EIS), the diameter of semicircle curves corresponded to the interfacial charge-transfer resistance (*R*_ct_), which controlled the electron transfer kinetics of the redox species at the electrode interface^25^. The obviously smaller EIS semicircle diameter of DAS(50%)-DNS/LDHs in light confirmed that the electron conductivity was augmented (Fig. 3C inset). However, the single-intercalated DAS/LDHs and DNS/LDHs photoanode were electric insulative without the EIS semicircle no matter in dark or in light. And the EIS semicircle diameters of other ratios for DAS(*x*%)-DNS/LDHs were diminished to some extent in light (Fig. S12), which was consistent with the *I-V* curves in light (Fig. 3B). Therefore, It could be concluded that the 2D PET process appeared in other DAS(*x*%)-DNS/LDHs composite; nevertheless, the ratio for the most efficient 2D PET process was 50%. This was also an obvious evidence of 2D photo-induced charge separation and transfer process within the interlayers of Zn_2_Al-LDHs. The DAS(*x*%)-DNS/LDHs photoanode exhibited similar IPCE spectra from 350 to 700 nm, and the IPCE values monotonically increased with the blue shift of incident wavelength that was consistent with the absorption spectra (Fig. 2A)^26^. In particular, the apparent IPCE value of DAS(50%)-DNS/LDHs was as high as 1.31% at 365 nm, and then fell to 0.55% at 700 nm (Fig. 3D), which was much higher than that of PTCBI/CoPc bilayer with IPCE under 0.5% (400–750 nm)^15^. For other ratios otherwise, these values were all less than that of the 50% composite, which demonstrated that the DAS/DNS anions with equal amount in the interlayers was the most efficient ratio for the 2D PET process. The high IPCE value of this co-intercalated composite was similar with the organic PEC water splitting materials (1.2%)^27^, which indicated the high performance for the 2D confined PET process between the co-intercalated DAS/DNS anions within the interlayers of LDHs.

Besides the activity of water splitting, structural and electrochemical stability is another significant criterion to evaluate an advanced photoelectron catalyst. The structural stability of photoanode before and after photo-electrochemical process was characterized by SEM and HRTEM. The surface morphology of DAS(50%)-DNS/LDHs remained unchanged after photoelectrochemical water splitting. Moreover, the HRTEM image of pristine DAS(50%)-DNS/LDHs nanosheets exhibited well-defined (110) lattice fringe, and hexagonally arranged spots^28^ in FFT pattern (Fig. S13) also remained after the PEC water splitting, which confirmed the crystallization stability of the co-intercalated system for the PEC water splitting. To investigate its electrochemical stability, long-term cycling stability of DAS(50%)-DNS/LDHs was investigated by performing continuous cyclic voltammetry (CV) between −0.1 and 0.8 V vs. SCE at 50 mV·s^−1^. As shown in Fig. S14A, a negligible difference can be observed between the curves measured at the initial cycle and that after 100 CV cycles, suggesting the excellent electrochemical durability of DAS(50%)-DNS/LDHs composite during the long-term CV cycling. When a constant potential of 0.8 V was applied, a continuous PEC water splitting process occurred to generate molecular hydrogen and oxygen. As shown in Fig. S14B, the current density exhibited a slight degradation even after a long period of 1200 s, which might be caused by the consumption of water or the accumulation of H_2_ and O_2_ on the electrode surface that hindered the reaction^29^. Thus, the stability under continuous water splitting process was identified for DAS(50%)-DNS/LDHs. This excellent long-term electrochemical and photo-stability was not surprised because of the remarkable UV stability of DAS and DNS (they are industrial fluorescent brighteners) in addition to the UV screen ability of Zn_2_Al-LDHs.

## Discussion

**Figure 4:**
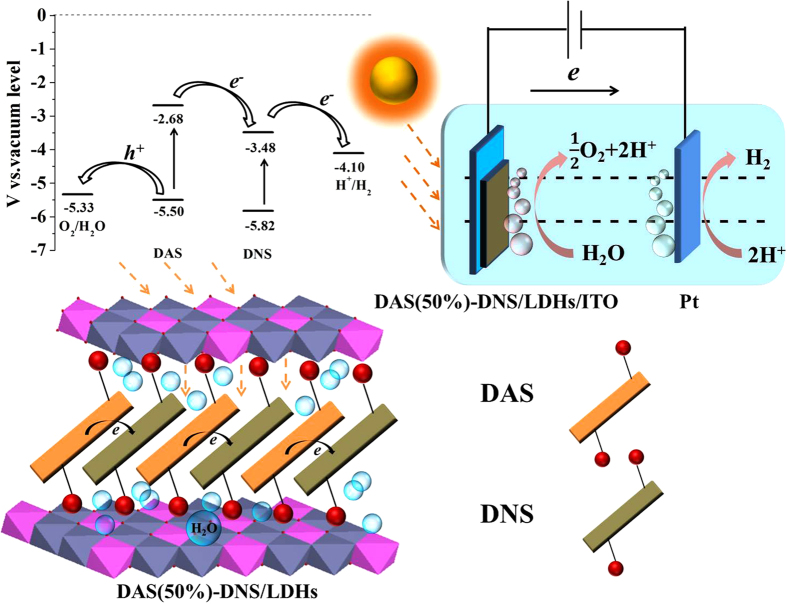
The proposed 2D PET mechanism scheme of photo-generated current of DAS(50%)-DNS/LDHs photoanode and the energy level alignment of DAS and DNS anions in Zn_2_Al-LDHs interlayers , E°(H^+^/H_2_) = 0–0.059 pH (vs. NHE), pH = 6.8, E°(O_2_/H_2_O) = 1.23–0.059 pH (vs. NHE), pH = 6.8 .

The proposed 2D PET mechanism scheme and the energy level alignment was illustrated in Fig. 4, which indicated that the photo-generated electron would transfer from DAS to DNS anions. The 2D PET process was realized within the interlayers, which resulted in the charge separation in the photoanode, and the free electrons reached the Pt cathode to reduce the proton to hydrogen, while the O_2_ was evolved at the photoanode by H_2_O molecules oxidized by the hole of the photoexcited DAS, and completed the whole PEC water splitting.

In summary, a simple and inexpensive of DAS(*x*%)-DNS/LDHs co-intercalated composites with broad optical absorption in UV-visible light region were successfully prepared. The co-intercalated DAS/DNS anions was orderly aligned within the interlayers and the HOMO/LUMO energy levels of the intercalated DAS and DNS anions were affected to match and couple as the electron donor and acceptor, respectively. The DAS(50%)-DNS/LDHs showed the remarkable PET performance with the 1.31% IPCE at 365 nm and enhanced photo-generated current (4.67 mA/cm^2^ at 0.8 V vs. SCE) for water splitting under UV-visible-light illumination with excellent crystalline, electrochemical and photo-stability. The DAS(50%)-DNS/LDHs composite exhibited the obvious 2D photo-induced charge separation and transfer process, which was a novel prototype for solar energy conversion. Further in-depth study was underway including designing the other suitable D/A pairs for optimizing the PEC water splitting, and improving the IPCE efficiency and stability. This novel inorganic/organic composite photoanode exhibited potential application prospect in PEC water splitting, and the flexibility of organic D/A pair selection and co-intercalation design for this inorganic/organic hybrid composite paved a broad and promising way to develop a kind of new, low-cost, and simple layered PEC system for solar energy conversion and application.

## Methods

### Materials

4,4-diaminostilbene-2,2-disulfonic acid (C_14_H_14_N_2_O_6_S_2_, DAS) and 4,4-dinitrostilbene-2,2-disulfonic acid (C_14_H_10_N_2_O_6_S_2_, DNS) were supplied by J&K Chemical Co. Ltd. Zn(NO_3_)_2_·6H_2_O, Al(NO_3_)_3_·9H_2_O, Na_2_SO_4_, and NaOH of analytical grades were purchased from Beijing Chemical Factory. The other ingredients were also analytical grade and used as received without further purification. Deionized/deCO_2_ water was used throughout the experimental process.

### Preparation of DAS(*x*%)-DNS/LDHs Composites

The DAS(*x*%)-DNS/LDHs powder was synthesized by the co-precipitation method reported previously. Zn(NO_3_)_2_·6H_2_O (0.01 mol) and Al(NO_3_)_3_·9H_2_O (0.005 mol) were dissolved in deionized/deCO_2_ water as solution A, DAS (*a* mol), DNS (*b* mol, in which *a* + *b* = 0.0025 mol, *x* % = *a*/(*a* + *b*), *x* % = 0, 10, 30, 50, 70, 90, and 100%, respectively.) and NaOH (0.03 mol) were dissolved in deionized/deCO_2_ water as solution B. Then the solution B was added into solution A with the dropping rate of 10 d/min under the protection of N_2_ atmosphere. Then the pH was adjusted into 7–8 with 0.1 M NaOH solution. The precipitates were developed immediately and then were aged in 100 ml PTFE-lined autoclave at 110 °C for 20 h. Then the powder products were obtained by centrifugation, washing, and vacuum drying.

### Fabrication of DAS(*x*%)-DNS/LDHs Thin Films

The DAS(*x*%)-DNS/LDHs composite thin film was prepared by the spin coating method. The suspension of the DAS(*x*%)-DNS/LDHs composite in ethanol (1 mg/mL) was thoroughly dispersed by an ultrasonicator under a N_2_ atmosphere for 15 min. After filtration using a membrane filter (0.2 μm, Millipore), 5 mL of the DAS(*x*%)-DNS/LDHs ethanol suspension was spin coated onto ITO coated glass substrate to obtain the thin films.

### Characterization

X-ray diffraction patterns (XRD) of the DAS(*x*%)-DNS/LDHs composites were recorded by a Shimadzu XRD-6000 diffractometer under the conditions: 40 kV, 50 mA, Cu Kα radiation (λ = 0.154056 nm) with step-scanned mode in step of 0.04°/2θ in the range from 3 to 70°, and a count time of 10 s/step. The Fourier transform infrared (FT-IR) spectra were recorded by a Nicolet 605 XB FT-IR spectrometer in the range 4000–400 cm^−1^ with 4 cm^−1^ resolution in air. The standard KBr disk method (1 mg of sample in 100 mg of KBr) was used. The morphology of thin films was investigated by using a scanning electron microscope (SEM; Hitachi S-4800) and the accelerating voltage applied was 5 kV. The surface roughness and thickness data were obtained by using the atomic force microscopy (AFM) software (Digital Instruments, Version 6.12). The solid-state UV-visible diffuse reflectance spectra were collected on a Shimadzu UV-3600 spectrophotometer in the range from 220 to 800 nm equipped with an integrating sphere attachment using BaSO_4_ as reference, and the slit width of 5.0 nm. The fluorescence spectra were collected at room temperature on a Hitachi F-7000 fluorescence spectrophotometer with the excitation and emission slit were set to 5 nm. The film was fabricated by spin coating method on KW-4A spin coater. High-resolution transmission electron microscopy (HRTEM) images were recorded with JEOL JEM-2100F field emission electron microscope. Dissolved oxygen was measured by METTLER SevenGo ProTM DO SG6. The pH value was measured by Sartorius basic pH Meter PB-10.

### PEC Experiments

All photoelectrochemical studies were operated on an electrochemical workstation (CHI 660E, CH Instruments Inc., Shanghai, China) in a home-built three-electrode optical cell using SCE as the reference electrode and a Pt wire as the counter electrode. Measurements were performed in a 0.5 M Na_2_SO_4_ solution (pH 6.8, purged with N_2_ for 30 min.) as the supporting electrolyte medium. The series of DAS(*x*%)-DNS/LDHs composites were fabricated as photoanode on the ITO coated glass substrate with an area of ~2 cm^2^, respectively. The water splitting photoelectrode was illuminated at 100 mW·cm^−2^ from a 150 W xenon lamp. Amperometric *I*–*t* curves of DAS(*x*%)-DNS/LDHs were recorded at an applied voltage of +0.8 V at 100 mW·cm^−2^. The sample was illuminated from the back side of the photoanode at room temperature.


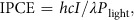


where *h* is the Planck’s constant, *c* is the speed of light, *I* is the photocurrent density (mA/cm^2^); λ is the incident light wavelength (nm), and P_light_ (mW·cm^−2^) is the power density of monochromatic light at a specific wavelength.

## Additional Information

**How to cite this article**: Zheng, S. *et al.* An Inexpensive Co-Intercalated Layered Double Hydroxide Composite with Electron Donor-Acceptor Character for Photoelectrochemical Water Splitting. *Sci. Rep.* **5**, 12170; doi: 10.1038/srep12170 (2015).

## Supplementary Material

Supplementary Information

## Figures and Tables

**Table 1:** The measured electrode potentials and energy levels of the DAS/LDHs, DNS/LDHs, DAS, and DNS.

	***E*_ox_^a^ (V)**	***E*_HOMO_^b^ (eV)**	***E*_LUMO_^c^ (eV)**	***E*_g_^d^ (eV)**
DAS/LDHs	0.76	−5.50	−2.68	2.82
DNS/LDHs	1.08	−5.82	−3.48	2.34
DAS	0.63	−5.37	−2.27	3.10
DNS	1.03	−5.77	−2.83	2.94

^a^Oxidation potentials measured by cyclic voltammetry.

^b^*E*_HOMO_ (eV) = −4.74 − *eE*_ox_.

^d^*E*_LUMO_ = *E*_HOMO_ + *E*_g_.

^c^The band gap (*E*_g_) was estimated from the UV-vis. absorption spectra and PL emission spectra.
