# Supplementary material for: An Inexpensive Co-Intercalated Layered Double Hydroxide Composite with Electron Donor-Acceptor Character for Photoelectrochemical Water Splitting
Source: Sci Rep. 2015 Jul 15;5:12170. doi: 10.1038/srep12170 (PMC4502407; doi:10.1038/srep12170)
Supplement: Supplementary Information [file srep12170-s1.doc]

**Supporting Information**

**A Inexpensive Co-Intercalated Layered Double Hydroxide Composite with Electron Donor-Acceptor Character for Photoelectrochemical Water Splitting**

Shufang Zheng^[a][c]^, Jun Lu*^[a][c]^, Dongpeng Yan^[b]^, Yumei Qin^[a]^, Hailong Li^[a]^, David G. Evans^[a]^ & Xue Duan^[a]^

^[a]^ State Key Laboratory of Chemical Resource Engineering, Beijing University of Chemical Technology, 15 Beisanhuan East Road, P. Box 98, 100029, Beijing (P. R. China)

E-mail: lujun@mail.buct.edu.cn

^[b]^ College of Chemistry, Beijing Normal University, 19 Xinjiekou Outside Street, 100875, Beijing (P. R. China)

^[c]^ Beijing Engineering Center for Hierarchical Catalysts, Beijing University of Chemical Technology, 15 Beisanhuan East Road, P. Box 98, 100029, Beijing (P. R. China)

**List of Contents**

**1. Structural characterization of** **DAS (*x*%)-DNS/LDHs**

**Fig. S1** The XRD patterns of the as-prepared samples, DAS (*x*%)-DNS/LDHs.

**Fig. S2** The layer spacing of DAS(*x*%)-DNS/LDHs.

**Fig. S3** The co-intercalation model of DAS and DNS within the interlayers of Zn_2_Al-LDHs.

**Fig. S4** FT-IR spectra of DAS(*x*%)-DNS/LDHs.

**Fig. S5** FT-IR spectra of DNS and DAS powders.

**2. Optical absorption and fluorescence spectra of DAS (*x*%)-DNS/LDHs**

**Fig. S6** Digital photos of DAS(*x*%)-DNS/LDHs powders.

**Fig. S7** The UV-vis. absorption spectra (A), and The PL emission spectra (B) of mixed aqueous solutions of DAS and DNS (*λ_ex_* = 280 nm).

**Fig. S8** The PL excitation and emission spectra of DAS (10^-3^ M) (dash line) and DNS (10^-2^ M) solution (solid line).

**3. Analysis of energy level of DAS/DNS, and PEC properties of DAS(*x*%)-DNS/LDHs**

**Fig. S9** (A) Cyclic voltammograms curves of DAS/LDHs(solid line) and DNS/LDHs(dash line) powders, SCE: standard electrode -4.74 eV vs. vacuum; (B) The diffuse reflectance spectra and photoemission spectra of DAS/LDHs (solid line) and DNS/LDHs (dash line) powders.

**Fig. S10** (A) Cycling voltammetry curves of DAS (10^-2^ M,solid line) and DNS (10^-2^ M, dash line) solution; (B) UV-vis absorption and PL spectra of DAS (10^-2^ M, solid line) and DNS (10^-2^ M, dash line).

**Fig. S11** The product detection of the PEC water splitting with DAS(50%)-DNS/LDH as the photoanode. (A) The pH value near the Pt cathode; (B) The dissolved oxygen measurement near the photoanode.

**Fig. S12** The electrochemical impedance spectra of DAS(10%)-DNS/LDHs(A), DAS(30%)-DNS/LDHs(B), DAS(70%)-DNS/LDHs(C), DAS(90%)-DNS/LDHs(D) photoanode under 300W Xe lamp irradiation.

**Fig. S13** The SEM (up) and HRTEM (down, inset: the corresponding FFT pattern) images of the DAS

(50%)-DNS/LDHs (A, C)before and (B, D) after photoelectrochemical water splitting.

**Fig. S14** (A) The cycling stability of DAS(50%)-DNS/LDHs at 0.8V vs. SCE (Insert: Current-voltage curves revealing that water splitting activity was observed for DAS(50%)-DNS/LDHs even after 100 CV cycles); (B) Time dependence of the current density at 0.8V vs. SCE for DAS(50%)-DNS/LDHs.

**1. Structural characterization of** **DAS(*x*%)-DNS/LDHs**

**
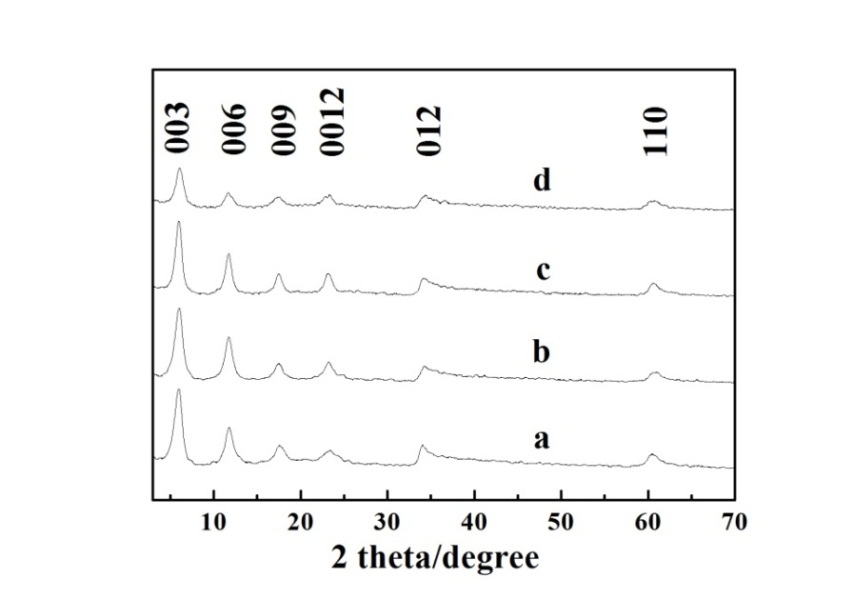
**

**Fig. S1** The XRD patterns of the as-prepared samples, a-DAS(10%)-DNS/LDHs; b-DAS(30%)-DNS/LDHs; c-DAS(70%)-DNS/LDHs; d-DAS(90%)-DNS/LDHs.

Generally, the 003, 006, 009 diffraction peaks are the characteristic in the X-ray diffraction pattern of NO_3_-Zn_2_Al-LDHs. All the intercalating products presented this characteristic diffraction peaks of 00*l* as shown in Fig. 1A and Fig. S1 in SI, and the location of 003 shifted to the smaller angle compared with that of NO_3_-LDHs, which indicated the successful intercalation of DAS/DNS anions.

**
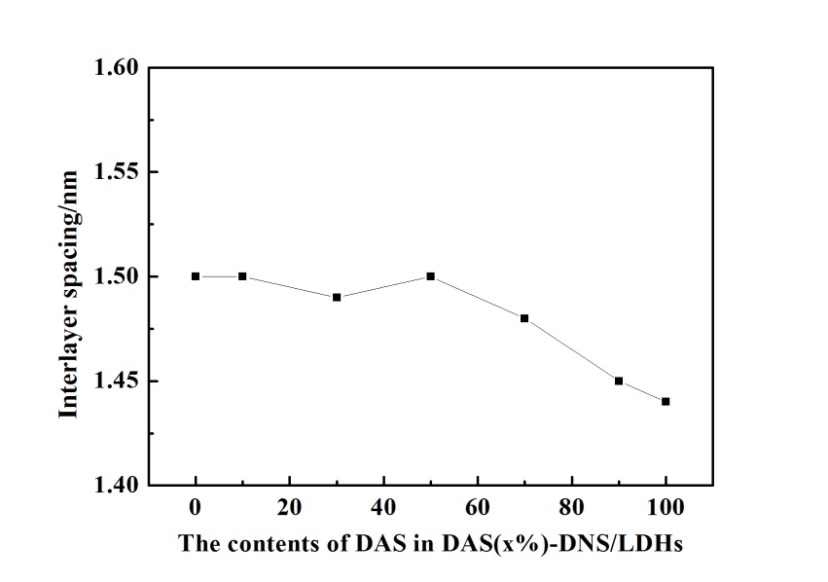
**

**Fig. S2** The layer spacing of DAS(*x*%)-DNS/LDHs.


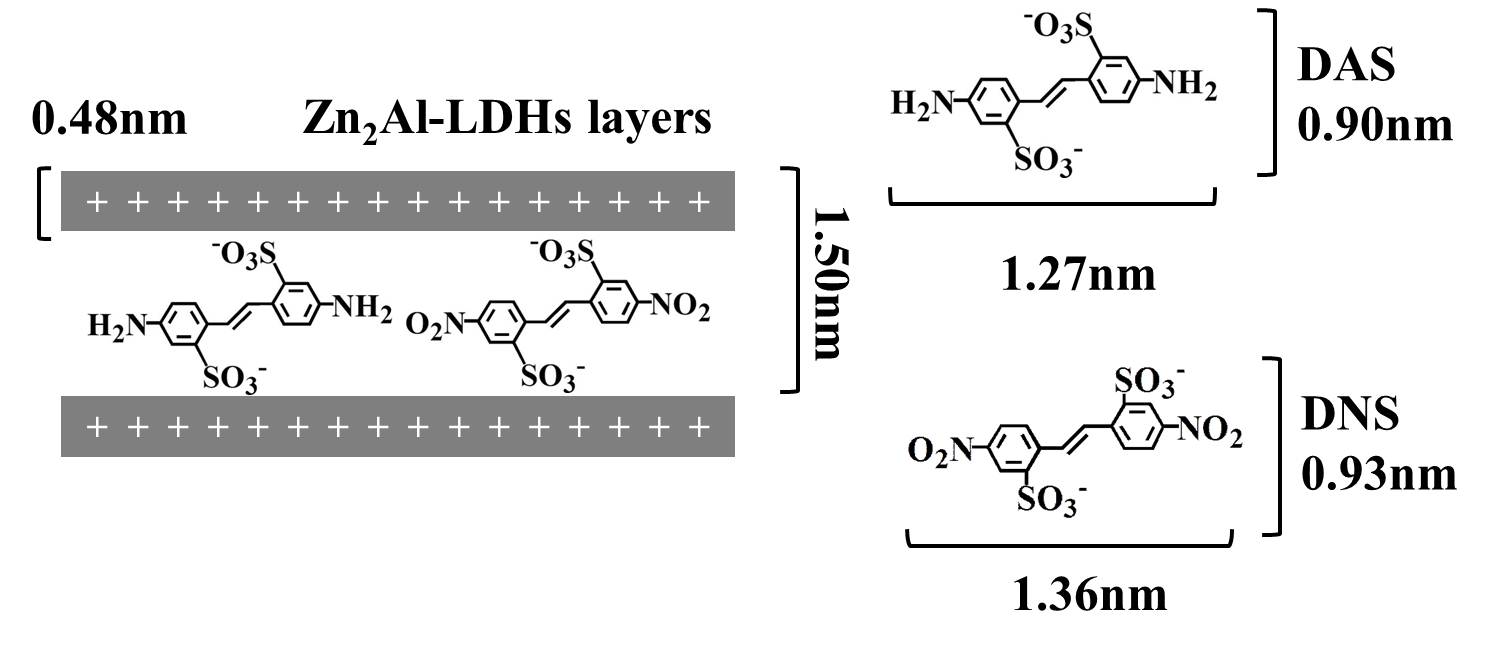


**Fig. S3** The co-intercalation model of DAS and DNS within the interlayers of Zn_2_Al-LDHs.


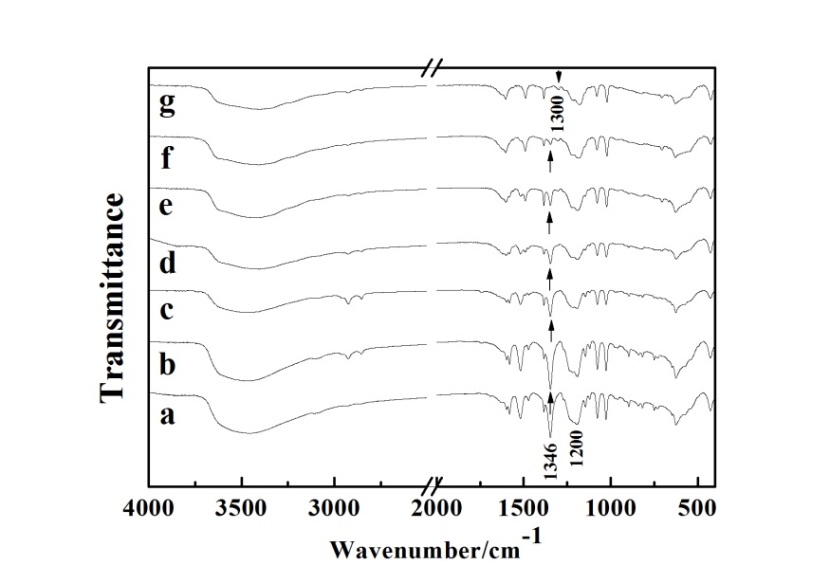


**Fig. S4** FT-IR spectra, a-DNS/LDHs; b-DAS(10%)-DNS/LDHs; c-DAS(30%)-DNS/LDHs; d-DAS (50%)-DNS/LDHs; e-DAS(70%)-DNS/LDHs; f-DAS(90%)-DNS/LDHs; g-DAS/LDHs.

*
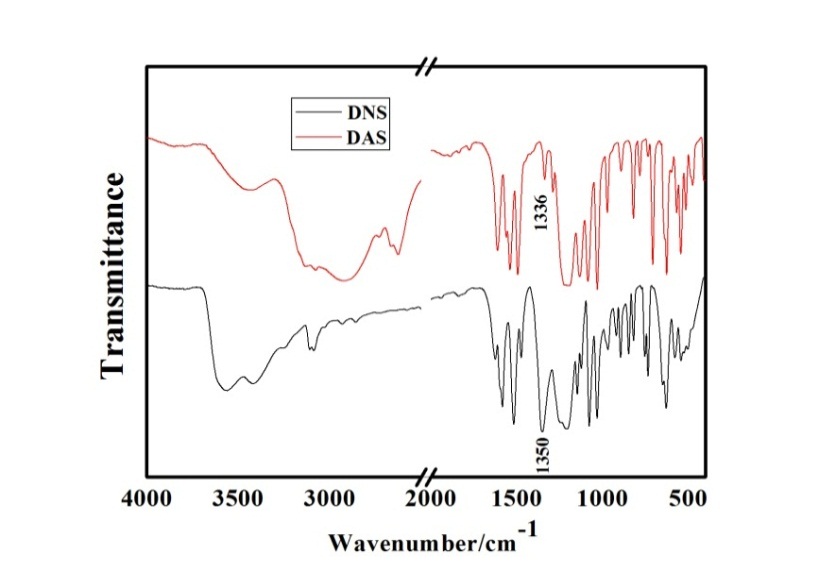
*

**Fig. S5** FT-IR spectra of DNS and DAS powders.

Although DAS and DNS tethered the different functional groups, due to their structural analogy, they arranged similarly within the interlayers of LDHs. FT-IR was adopted to further characterize the co-intercalating system. As shown in Fig. S4, all the FT-IR curves exhibited the characteristic absorption peak of -SO_3_ at 1200 cm^-1^. The 1346 cm^-1^ was the characteristic peak of-NO_2_^-^ according to the IR spectra of DNS (Fig. S5). In curve a, the appearance of peak at 1200 cm^-1^ and 1346 cm^-1^ indicated that the DNS anions were successfully intercalated into LDHs layers, Similarly, in curve g, the peak at 1200 cm^-1^ was the evidence of successful intercalation of DAS anion, and the small peaks of amino groups at 1300 cm^-1^ pointed the presence of -NH_2_ according to the IR spectra of DAS (Fig. S5). However, in the curves b-f, the smaller sharp peak of nitro groups was attributed to the content variation of DNS within the interlayers.

**2. Optical absorption and fluorescence spectra of DAS (x%)-DNS/LDHs**


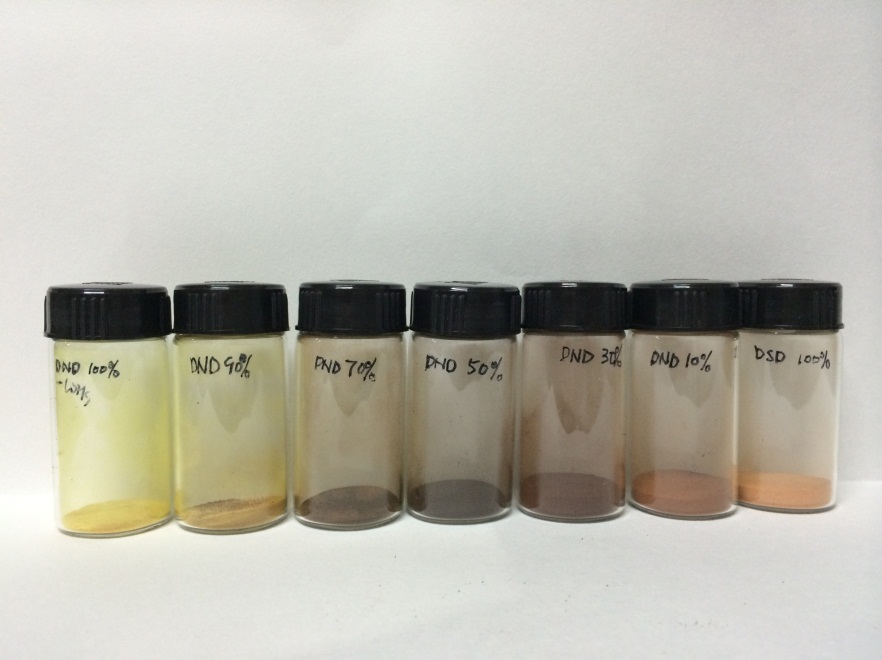


**Fig. S6** Digital photos of DAS(*x*%)-DNS/LDHs powders (*x* is 0, 10, 30, 50, 70, 90, 100 from left to right).

**
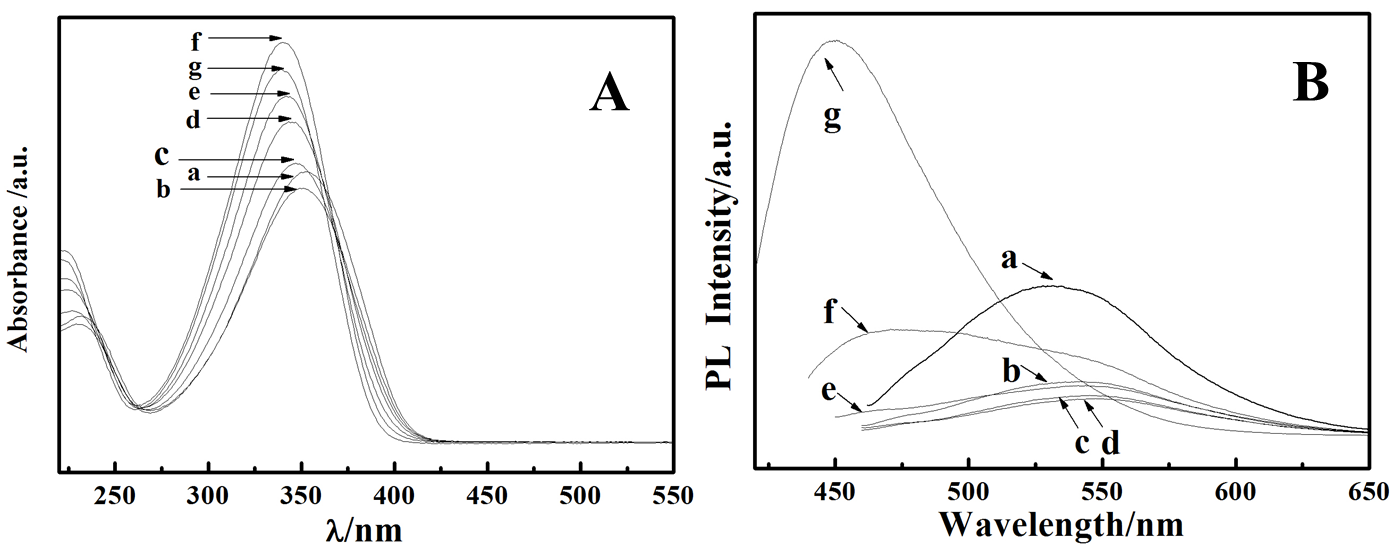
**

**Fig. S7** The UV-vis. absorption spectra (A), and The PL emission spectra (B) of mixed aqueous solutions of DAS (*x*%) and DNS (a) 0%, (b) 10%, (c) 30%, (d) 50%, (e) 70%, (f) 90%, (g) 100% (*λ_ex_* = 280 nm).


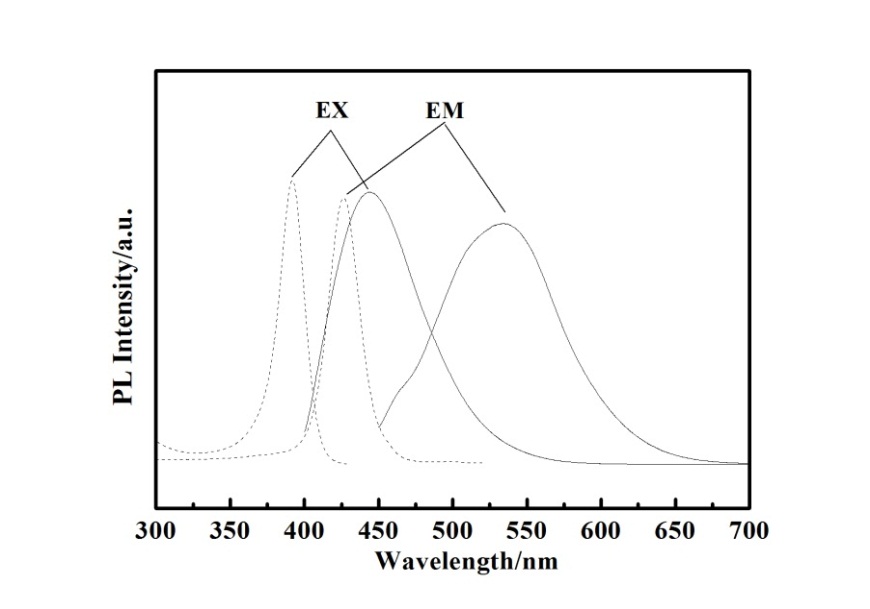


**Fig. S8** The PL excitation and emission spectra of DAS (10^-3^M) (dash line) and DNS (10^-2^M) solution (solid line).

**3. Analysis of energy level of DAS/DNS, and PEC properties of DAS(x%)-DNS/LDHs**


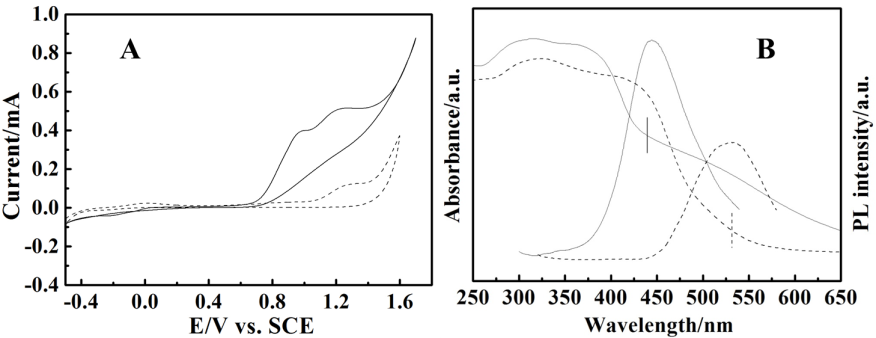


**Fig. S9** (A) Cyclic voltammograms curves of DAS/LDHs(solid line) and DNS/LDHs(dash line) powders, SCE: standard electrode -4.74 eV vs. vacuum; (B) The diffuse reflectance spectra and photoemission spectra of DAS/LDHs (solid line) and DNS/LDHs (dash line) powders.

**
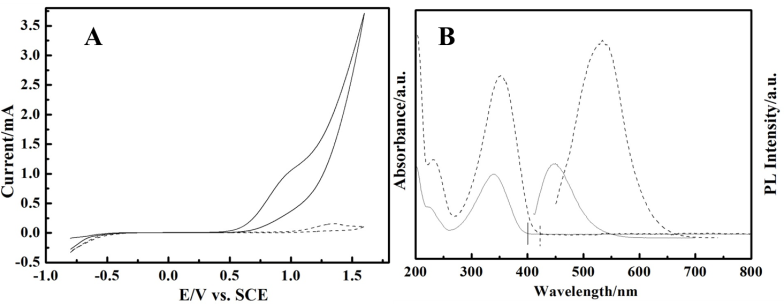
**

**Fig. S10** (A) Cycling voltammetry curves of DAS (10^-2^ M,solid line) and DNS (10^-2^ M, dash line) solution; (B) UV-vis absorption and PL spectra of DAS (10^-2^ M, solid line) and DNS (10^-2^ M, dash line).


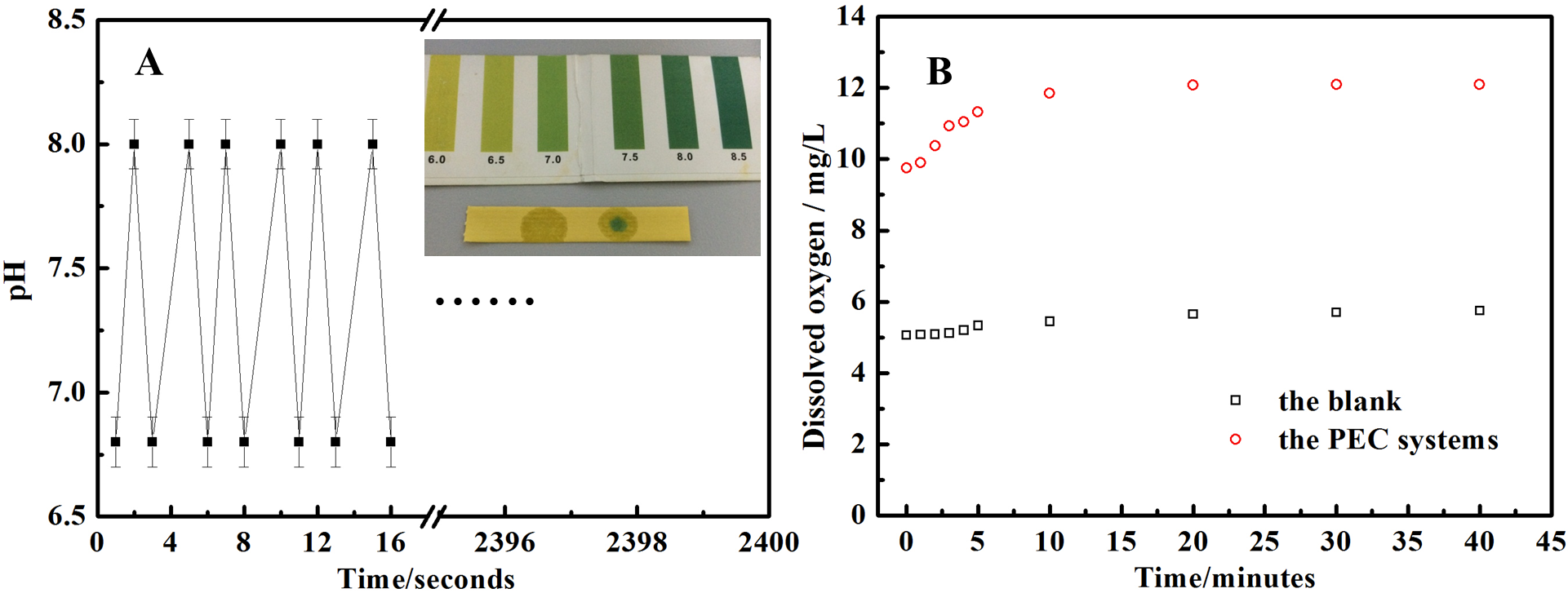


**Fig. S11** The product detection of the PEC water splitting with DAS(50%)-DNS/LDH as the photoanode (A) The pH value near the Pt cathode; (B) The dissolved oxygen measurement near the photoanode.

We have detected the hydrogen evolving reaction by the changes of pH near the Pt cathode, and the oxygen evolving reaction by the amount of dissolved oxygen. As shown in Fig. S11 A, the pH value was increased from 6.8 to 8.0 after 1-2 minutes due to the H_2_ evolution, and restored to 6.8 immediately due to the diffusion of H^+^ from the bulk solution. Therefore, the changes in pH value were continuous and periodic throughout the whole process of PEC water splitting. In addition, the amount of dissolved O_2_ was increased to 12 mg/mL (approaching the saturated concentration of dissolved O_2_ at 20°C) within 10 minutes; that was a direct indication of the evolution of O_2_ (Fig. S11 B). Therefore, we conclude that the PEC water splitting was realized with the co-intercalated LDHs as photoanode.


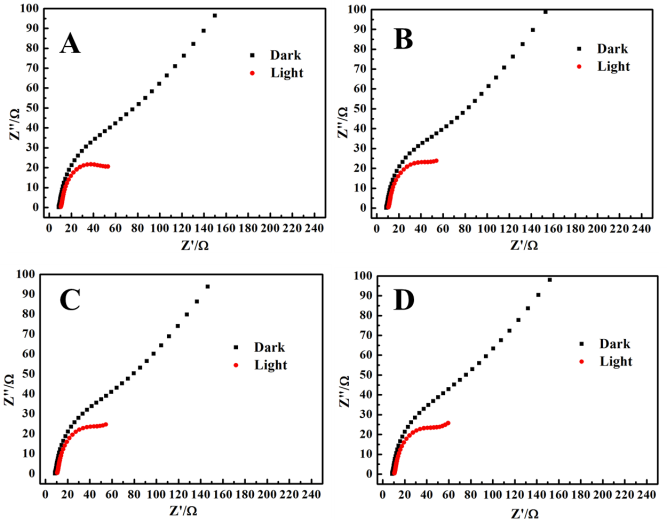


**Fig. S12** The electrochemical impedance spectra of DAS(10%)-DNS/LDHs (A), DAS(30%)-DNS/LDHs (B), DAS(70%)-DNS/LDHs (C), DAS(90%)-DNS/LDHs (D) photoanode under 300W Xe lamp irradiation.


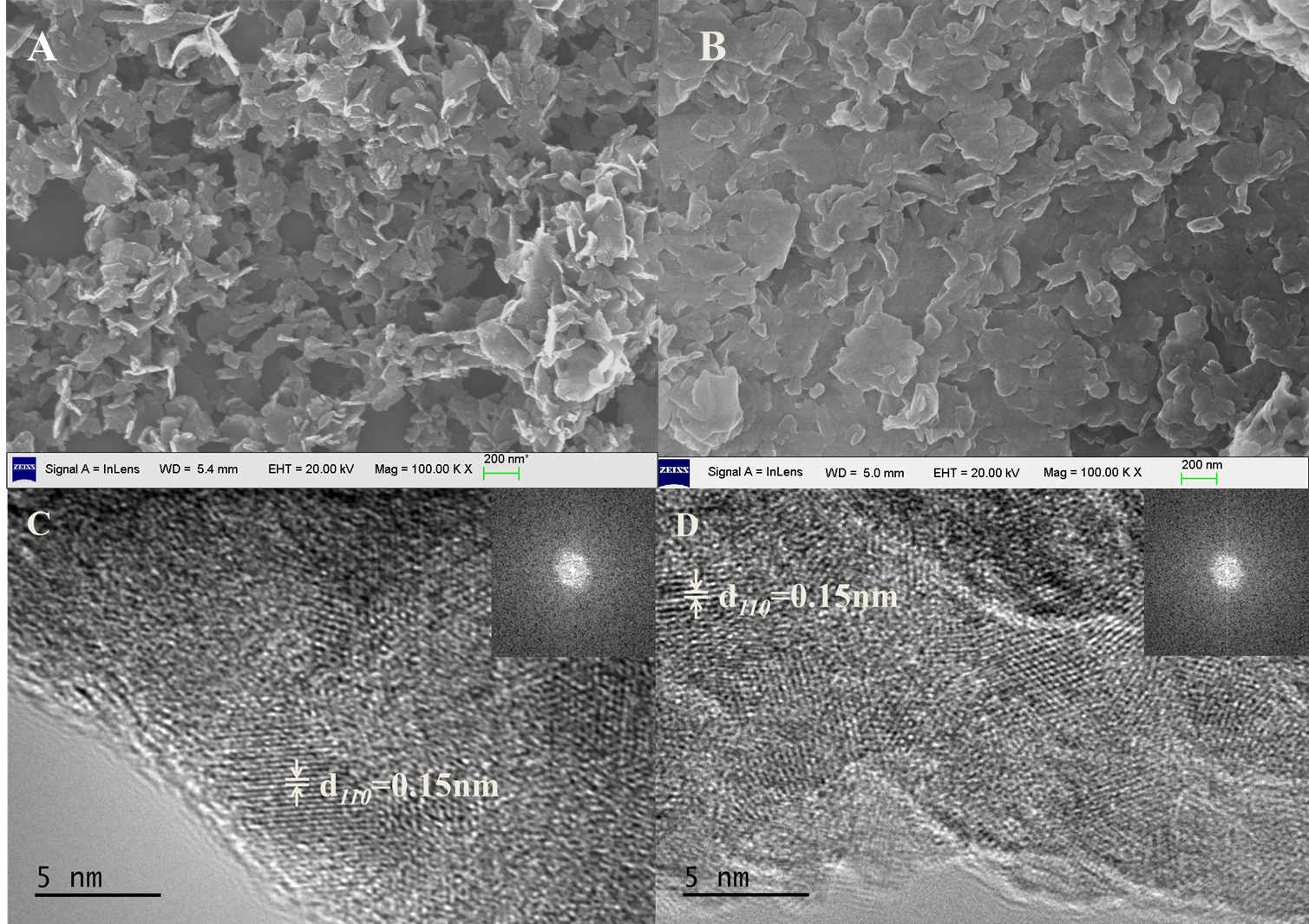


**Fig. S13** The SEM (up) and HRTEM (down, inset: the corresponding FFT pattern) images of the DAS (50%)-DNS/LDHs (A, C) before and (B, D) after the photoelectrochemical water splitting


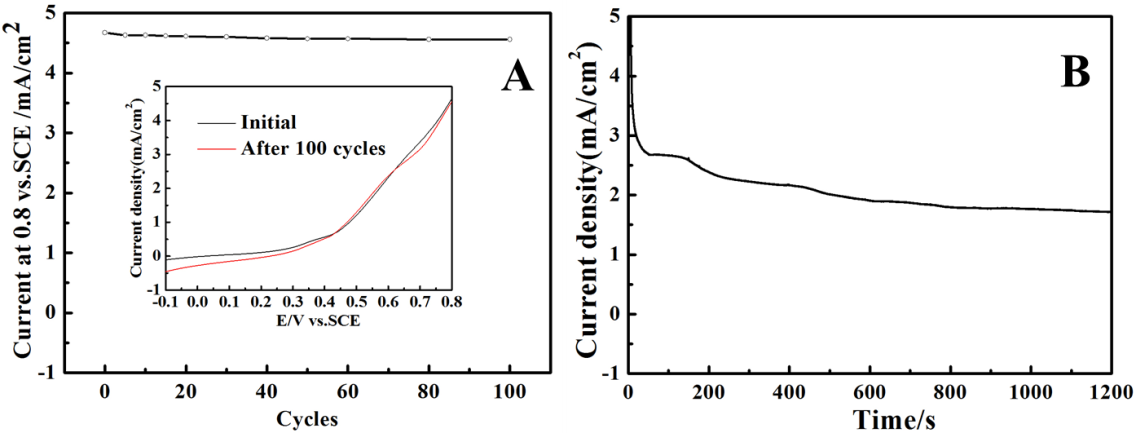


**Fig. S14** (A) The cycling stability of DAS(50%)-DNS/LDHs at 0.8V vs. SCE (Insert: Current-voltage curves revealing that water splitting activity was observed for DAS(50%)-DNS/LDHs even after 100 CV cycles). (B) Time dependence of the current density at 0.8V vs. SCE for DAS(50%)-DNS/LDHs.
